# Systematic analysis of the clinical and biochemical characteristics of maternally inherited hypertension in Chinese Han families associated with mitochondrial

**DOI:** 10.1186/s12920-014-0073-x

**Published:** 2014-12-24

**Authors:** Yuqi Liu, Qinglei Zhu, Chao Zhu, Xueping Wang, Jie Yang, Tong Yin, Jinliao Gao, Zongbin Li, Qinghua Ma, Minxin Guan, Yang Li, Yundai Chen

**Affiliations:** Cardiology Department, Chinese PLA General Hospital, Beijing, China; Attardi Institute of Mitochondrial Biomedicine and Zhejiang Provincial Key Laboratory of Medical Genetics, Wenzhou Medical College, Wenzhou, Zhejiang China; Division of Human Genetics, Cincinnati Children’s Hospital Medical Center, Cincinnati, OH USA; Department of Genetics, College of Life Sciences, Zhejiang University, Hangzhou, Zhejiang China; Department of Cardiology, Yishui Center Hospital of Shandong Province, Yishui, Shandong China; Cardiology Department of General Hospital of People’s Liberation Army, Institute of Geriatric Cardiology, No. 28 Fuxing Road, Hai dian District, Beijing, 100853 PR of China

**Keywords:** mitochondrial DNA, Hypertension, Chinese, Mutations, Maternal

## Abstract

**Background:**

Mitochondrial DNA mutations may be associated with cardiovascular disease, including the common cardiac vascular disease, hypertension.

**Methods:**

In this study we performed segregation analysis and systematically evaluated the entire mitochondrial genome in nine maternally inherited hypertension probands from Chinese Han families. We also performed clinical, genetic and molecular characterization of 74 maternally inherited members from these families and 216 healthy controls.

**Results:**

In the maternally inherited members, 12 had coronary heart disease (CHD), six had cerebrovascular disease, five had diabetes, nine had hyperlipidemia and three had renal disease. Laboratory tests showed that the sodium and potassium levels in blood of the maternally inherited members were higher than those of the control group (*P* < 0.01), while no differences were observed in fasting blood glucose (FBG), total cholesterol (TC), triglyceride, low density lipoprotein cholesterol (LDL-c) and creatinine levels (*P* > 0.05). The high density lipoprotein cholesterol (HDL-c) level of the maternally inherited members was lower than that of the control group (*P* = 0.04). The whole mitochondrial DNA sequence analysis revealed a total of 172 base changes, including 17 in ribosomal RNA (rRNA) genes, four in transfer RNA (tRNA) genes, and 22 amino acid substitutions. The remainder were synonymous changes or were located in non-coding regions. We identified seven amino acid changes in the nine maternally inherited hypertension families, including four mutations in ATPase6 and three in Cytb. More interestingly, tRNA^Ser(UCN)^ 7492 T > C was absent in controls and was present in <1% of 2704 mtDNAs, indicating potential functional significance.

**Conclusions:**

This study showed that mutations in mtDNA may contribute to the pathogenesis of hypertension in these Chinese Han families. In the near future, identification of additional mtDNA mutations may indicate further candidate genes for hypertension.

**Electronic supplementary material:**

The online version of this article (doi:10.1186/s12920-014-0073-x) contains supplementary material, which is available to authorized users.

## Background

Hypertension is a major public health problem, affecting approximately 1 billion people worldwide [1]. Hypertension is also a major risk factor for coronary heart disease, stroke, congestive heart failure and renal disease [2]. Essential hypertension is commonly regarded as a multifactorial disease influenced by both genetic and environmental factors. Familial aggregation of high blood pressure, despite different environmental factors, suggests that genetic factors are involved in the etiology of hypertension [3,4]. Estimates of genetic variance range from 20% to 50% [5-7].

Early and more recent investigations [8,9] show significant maternal familial aggregation of high blood pressure, which suggests a contribution of the mitochondrial genome to hypertension [10]. Our previous studies have reported excess maternal transmission of hypertension (HTN) in hypertensive families associated with tRNA point mutations [11-15]. Here we report mtDNA mutations, including previously unreported mutations, in 108 hypertension patients and 216 healthy controls.

## Methods

### Subjects

As part of a genetic screening program for hypertension, 324 subjects, including 108 hypertension patients and 216 controls, were ascertained at the Institute of Geriatric Cardiology of the Chinese People’s Liberation Army (PLA) General Hospital. Control subjects underwent physical examination, provided family medical history, and provided samples for laboratory assessment at least twice in 1 year. Hypertension was defined according to the recommendations of the Joint National Committee on Detection, Evaluation and Treatment of High Blood Pressure (JNC VI) [16] as a systolic blood pressure of 140 mmHg or higher and/or a diastolic blood pressure of 90 mmHg or greater.

We performed segregation analysis on 108 hypertension patients to identify maternally inherited hypertension. We applied the following exclusion criteria: a. the proband’s father suffered from hypertension; b. if the proband was male, and one or more of his offspring suffered from hypertension; c. neither the proband’s mother nor her offspring presented with hypertension; d. the probands’ spouses presented with high blood pressure; e. inheritance was consistent with autosomal recessive, autosomal dominant, X-linked, and Y-linked patterns (see reference [17]). These criteria resulted in the exclusion of 99 probands. The other nine probands presented with a maternally inherited pattern. Their families (including a further 65 maternally inherited members) and 216 healthy controls were interviewed and evaluated to identify both personal or medical histories of hypertension and other clinical abnormalities. Medical history, including coronary heart disease (CHD), cerebrovascular disease, diabetes, hyperlipidemia and renal disease, was also evaluated. Patients reporting cigarette use within 1 year prior to examination were considered as smokers. Body mass index (BMI) (Kg/m^2^) is defined as an individual’s body mass divided by the square of their height. BMI of 18.5 to 25 indicates optimal weight. All patients had a standard 12-lead ECG recording at 25 mm/s and 1 mV/cm. Left ventricular hypertrophy (LVH) was assessed according to traditional Sokolow-Lyon voltage criteria (SV1 + RV5 or RV6 ≥ 3.5 mV) or to gender-specific Cornell voltage (RaVL + SV3 ≥ 2.8 mV in men or ≥2.0 mV in women) criteria. Total DNA samples of the 65 (non-proband) maternal members and 216 healthy controls were acquired for sequence analysis. Informed consent was obtained from all participating members.

All participating members are fully informed of the purpose of the study, the test items (including the physical examination, family medical history, and blood samples for laboratory assessment and sequencing analysis), the results of the laboratory assessment and sequencing analysis. All the patients were fully informed the study and signed the informed consent to join the study and consent to publish their individual data. All the protocols were approved by the ethics committee of the Chinese PLA General Hospital.

### mtDNA sequencing and sequence analysis

Genomic DNA was isolated from whole blood cells of participants using Puregene DNA Isolation Kits (Gentra Systems, Minneapolis, MN, USA). The entire mitochondrial genome of HTN subjects and controls was PCR-amplified in 24 overlapping fragments using light-strand and heavy-strand sets of oligonucleotide primers [18]. Each fragment was purified and subsequently analyzed by direct sequencing on an ABI 3700 automated DNA sequencer (Applied Biosystems, Inc., Foster City, CA, USA) using the Big Dye Terminator Cycle sequencing reaction kit. The resultant sequence data were compared with the revised consensus Cambridge sequence (GenBank accession No. NC-012920, http://www.mitomap.org/MITOMAP) [19]. All mtDNA mutations were individually analyzed using the MitoAnalyzer (National Institutes of Standards and Technology, Gaithersburg, MD, USA, http://www.cstl.nist.gov/biotech/strbase/mitoanalyzer.html) and the MITOMAP database [20]. The haplogroups were deduced by comparing the complete mtDNA sequence data with the previously reported haplogroup-specific variants [21]. To analyze the phylogeny of tRNAs, we used vertebrate mitochondrial DNA sequences for interspecific analysis, including from *Bos Taurus*, *Cebus albifrons*, *Gorilla gorilla*, *Homo sapiens*, *Hylobates lar, Lemur catta*, *Macaca mulatta*, *Macaca sylvanus*, *Mus musculus*, *Nycticebus coucang*, *Pan paniscus*, *Pan troglodytes*, *Papio hamadryas*, *Pongo abelii*, *Pongo pygmaeus*, *Tarsius bancanus*, and *Xenopus laevis* (GenBank). The conservation index (CI) was calculated by comparing the human nucleotide variants with the other 16 vertebrates. The CI was then defined as the percentage of species from the list of 17 vertebrates that have the wild-type nucleotide at that position.

### Statistical analysis

Statistical analyses were performed using the Statistical Package for Social Sciences software (SPSS version 13.0). Continuous variables with normal distributions were expressed as means ± SD and compared using a *t* test. Categorical variables were compared using the chi-squared test where appropriate.

## Results

### Clinical evaluation and inheritance analysis in nine families

**Figure 1:**
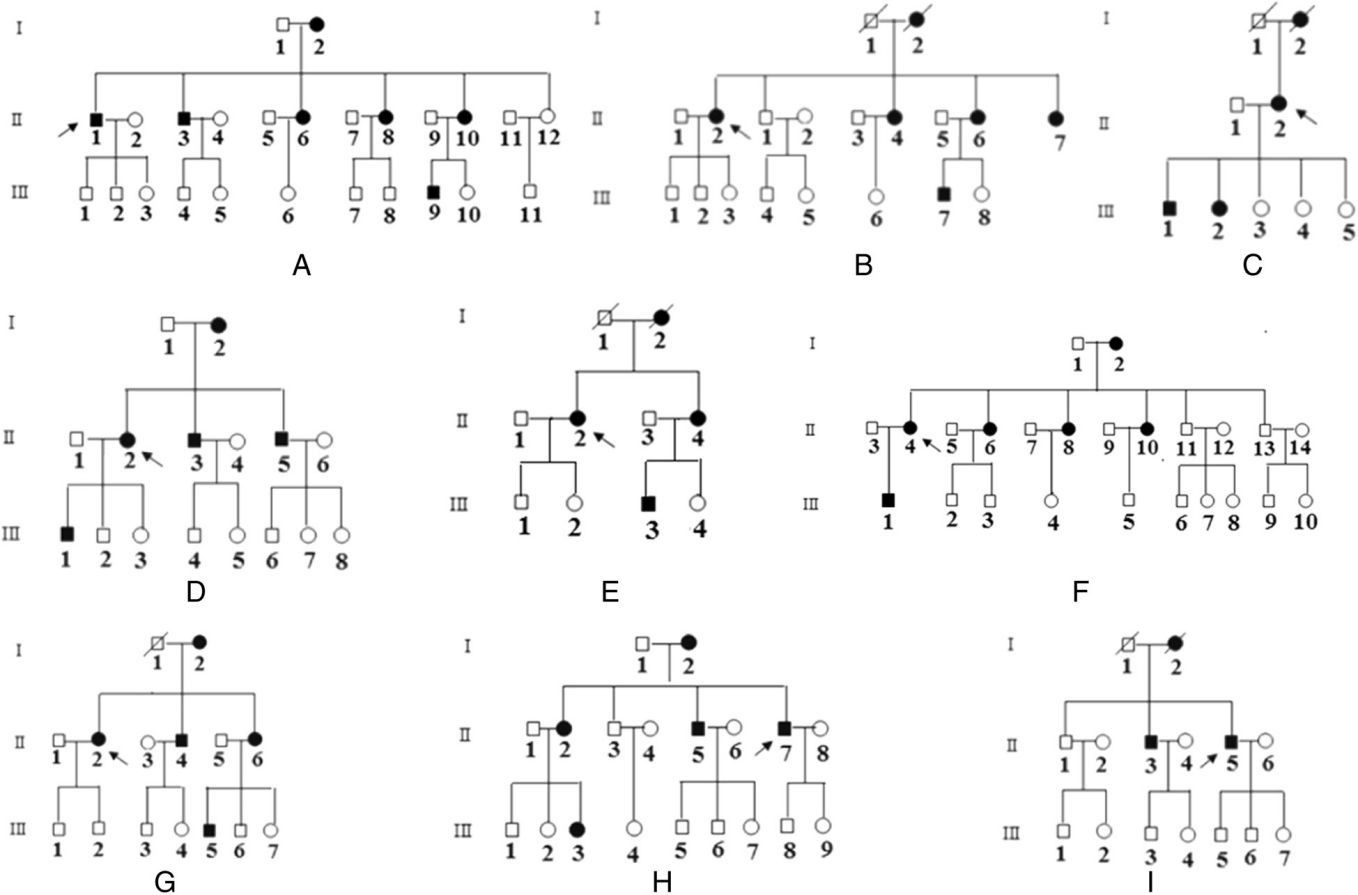
**Nine Chinese Han pedigrees with maternally inherited hypertension.** Affected individuals are indicated by filled symbols. Arrows denote probands. I means first generation; II means second generation; III means third generation.

As shown in Figure 1 and Table 1, there were three male probands (indicated by arrows), HTN-1 (Figure 1A), HTN-8 (Figure 1H) and HTN-9 (Figure 1I). All of their mothers suffered from high blood pressure, and none of their offspring had hypertension. One brother (II-3, Figure 1A) and three out of four sisters (II-6, 8, 10) of HTN-1 also had hypertension. HTN-8’s brother (II-5, Figure 1H) and one of two sisters (II-2) suffered from hypertension, but no offspring of HTN-8 or his brother presented with high blood pressure. Only one daughter of his sister (III-3) had hypertension. One out of two brothers (II-3) of HTN-9 had hypertension, but no offspring of HTN-9 or his brothers presented with high blood pressure. Similarly, we found that in the families of the female probands (HTN-2, 3, 4, 5, 6 and 7, Figure 1B, C, D, E, F, G) only the offspring of the affected female patients presented with hypertension. There was no evidence that any member of this family had any other cause to account for hypertension. Therefore, the inheritance pattern of all nine probands was consistent with maternal inheritance.

**Table 1:** **Summary of Clinical Data for 9 probands with HTN**

**Subjects**	**Gender**	**Age of Test (yrs)**	**Age of Onset (yrs)**	**Penetrance % (Maternal numbers)**	**Systolic Pressure (mm Hg)**	**Diastolic Pressure (mm Hg)**	**IVST, mm (6–12 mm)**	**LVMI (g/m** ^**2**^ **)**	**ECG**	**eGFR (ml/min/1.73 m** ^**2**^ **)**
HTN_1_	M	58	58	53.8(13)	160	80	9	87.2	N	125.3
HTN_2_*	F	62	53	50.0(12)	160	96	10	90.5	N	74.7
HTN_3_*	F	61	45	57.1(7)	154	88	13	112.3	LVH	117.7
HTN_4_*	F	40	33	71.4(7)	140	90	11	101.7	N	111.5
HTN_5_*	F	62	51	57.1(7)	160	80	12	92.7	N	130.1
HTN_6_*	F	56	56	50.0(12)	150	100	12	98.4	N	114.9
HTN_7_*	F	58	40	55.6(9)	180	100	13	91.2	N	65.3
HTN_8_*	M	52	30	62.5(8)	160	110	13	97.1	LVH	102.7
HTN_9_	M	75	72	75.0(4)	180	80	10	117.9	N	77.2

*These patients received antihypertension treatment. This table shows pretreatment blood pressures. eGFR indicates estimated glomerular filtration rate; F, female; IVST, interventricular septal thickness; LVH, ECG showed left ventricular hypertrophy; LVMI, left ventricular mass index; M, male; N, electrocardiography (ECG) was normal.

Of the nine probands, three were male patients in the age range of 40 to 75 years old. They came to the Chinese PLA General Hospital Cardiology Clinic for clinical evaluations. Onset-age ranged from 30 to 72 years old. Their blood pressure ranged from 140/90 to 180/100 mm Hg. HTN-3 and HTN-8 presented with LVH upon ECG examination. Echocardiography showed mild thickening of the interventricular septum in HTN-3, HTN-5, HTN-6, HTN-7 and HTN-8; however, this thickening did not reach the echocardiography standard for LVH diagnosis (male patient larger than 125 g/m^2^, female patient larger than 115 g/m^2^). We also calculated the estimated glomerular filtration rate (eGFRs) according to the abbreviated Modification of Diet in Renal Disease formula [22], and found that HTN-2, HTN-7 and HTN-9 had mild renal dysfunction with eGFRs lower than 90 mL/min/1.73 m^2^ (see Table 1).

We collected clinical data from the maternal family members of the nine probands (a total of 74 maternal family members) and 216 healthy controls (see Table 2). The average age was 60.9 ± 12.3 versus 55.9 ± 10.8 years for controls with no significant difference. The systolic blood pressure (SBP) and diastolic blood pressure (DBP) of the maternal group were higher than those of controls, 132.8 ± 29.6 versus 117.9 ± 9.8 and 79.3 ± 17.4 versus 66.8 ± 9.1, respectively, both *P* < 0.01. Twelve maternal members had CHD, six cerebrovascular disease, five diabetes, nine hyperlipidemia, and three renal disease. Laboratory tests showed no significant changes in FBG, TC, triglyceride, LDL-c and creatinine (*P* > 0.05). The sodium and potassium levels of the maternal family members were higher than those in controls (*P* < 0.01), while the HDL-c level was lower than that of controls (*P* = 0.04).

**Table 2:** **Cinical data for maternal members of the probands and the controls**

	**Maternal members (n = 74)**	**Controls (n = 216)**	***P*** **value**
	**Mean ± SD**	**Mean ± SD**	
Men, n (%)	28	52	0.07
Age (years)	60.9 ± 12.3	55.9 ± 10.8	0.15
Onset age(years)	45.6 ± 9.8		
BMI (kg/m^2^)	25.1 ± 4.8	24.1 ± 3.1	0.00*
Systolic BP (mmHg)	132.8 ± 29.6	117.9 ± 9.8	0.00*
Diastolic BP (mmHg)	79.3 ± 17.4	66.8 ± 9.1	0.00*
*Past disease*			
CHD	12	0	0.00*
Cerebrovacular disease	6	0	0.00*
Diabetes	5	0	0.00*
Hyerlipidemia	9	0	0.00*
Renal disease	3	0	0.02*
Alcohol	9	18	0.56
Current Smoking	18	21	0.01*
*Laboratory test*			
FSB (mmol/L)	5.7 ± 1.5	5.5 ± 1.8	0.38
TC (mmol/L)	4.3 ± 0.8	4.1 ± 1.0	0.11
Sodium (mmol/L)	141.2 ± 3.6	139.4 ± 3.2	0.00*
Potassium (mmol/L)	4.3 ± 0.4	4.1 ± 0.5	0.00*
Triglyceride (mmol/L)	1.7 ± 0.9	1.5 ± 0.8	0.07
HDL (mmol/L)	1.1 ± 0.3	1.2 ± 0.4	0.04*
LDL (mmol/L)	2.6 ± 0.6	2.5 ± 0.8	0.31
Creatinine (μmol/L)	61.4 ± 11.1	59.1 ± 15.8	0.23

CHD, coronary heart disease; FSB, fasting blood glucose; TC, total cholesterol; HDL, High-density lipoprotein; LDL, low-density lipoprotein; UN, Urea nitrogen.

* present statistically significant difference.

### Analysis of coding and control region mutations

We sequenced the entire mitochondrial genome in the nine probands. Twenty-four overlapping fragments, including 37 genes, were PCR-amplified, purified and subsequently analyzed by DNA sequencing. All variants were compared with the data in mitomap (http://www.mitomap.org) [20] and Phylotree (http://www.phylotree.org/). Comparison with the “revised Cambridge” reference sequence (rCRS) [19,23] revealed a total of 172 variants in the nine probands (see Additional file 1 and Table 3). All 172 variants identified in the probands were compared with the reference sequence and with corresponding sequence from 216 controls. Among the variants, 151 were located in the coding or control regions of genes, including 77 variants in the D-loop, four in ND1, five in ND2, seven in COI, six in COII, two in ATPase8, 13 in ATPase6, three in COIII, four in ND3, one in ND4L, five in ND4, seven in ND5, four in ND6 and 13 in Cytb. Twenty-four of the 151 variants were not identified in the controls or in 2704 control mtDNAs [24] and 22 variants were non-synonymous. To discover mutations associated with hypertension, we screened the other 65 maternally inherited members (74 minus the nine probands) for these 151 mutations. We identified COI 7028C > T and Cytb 15326A > G in all maternal members of HTN_2_, ND2 4769A > G, ATPase6 8584G > A and 8701A > G in HTN_3_, ATPase8 8413A > G in HTN_4_, D-loop 750A > G and ND5 12705C > T in HTN_5_, ATPase6 8803A > G in HTN_6_, COIII 9950C > T, Cytb 15662A > G and 15851A > G in HTN_7_, ATPase8 8413A > G, ATPase6 8794C > T and Cytb 15379C > T in HTN_8_, COIII 9380G > A and D-loop 16278C > T in HTN_9_. These changes include seven non-synonymous changes associated with amino acid substitutions.

**Table 3:** **All mtDNA variants in the nine probands with HTN**

**Location**	**Total variants**	**Num of Amino acid changes**	**New reported**
D-loop	78	-	24
12S rRNA	6	-	0
16S rRNA	11	-	5
ND1	4	0	1
tRNA^Gln^	1	-	0
ND2	5	2	2
COI	7	0	2
tRNA ^Ser(UCN)^	1	-	1
COII	6	0	0
ATPase8	2	1	0
ATPase6	13	6	6
COIII	3	0	0
ND3	4	2	0
ND4L	1	0	0
ND4	5	1	0
ND5	7	2	2
ND6	4	3	0
tRNA ^Glu^	1	-	1
Cytb	13	5	0
tRNA ^Thr^	1	0	0

See http//www.mitomap.org and http://www.genpat.uu.se/mtDB/ for more information.

### rRNA/tRNA mutation analysis

DNA fragments spanning the 12S rRNA, 16S rRNA and 22 tRNA genes were PCR-amplified from mitochondrial DNA of the nine probands. We identified 21 nucleotide changes, including six variants in the 12S rRNA gene, 11 in the 16S rRNA gene and four variants in four tRNA genes (see Additional file 1 and Table 3). There were six novel mutations, including 2448G > A, 2534G > A, 2673G > A, 2695G > A, 2706A > G in 16S rRNA and 14686G > A in tRNA^Glu^. All the nucleotide changes were verified by sequence analysis of both strands and appeared to be homoplasmic. We also identified the 21 mutations in the other 65 maternally inherited members. We identified 12S rRNA 1005 T > C, 16S rRNA 1824 T > C and tRNA^Ser(UCN)^ 7492C > T in all maternally inherited members of HTN_1_, 12S rRNA 1438A > G and 16S rRNA 2706A > G in HTN_2_, 12S rRNA 1438A > G and 16S rRNA 2706A > G in HTN_5_, tRNA^Thr^ 15927G > A in HTN_7_, and 16S rRNA 1736G > A in HTN_8_.

**Figure 2:**
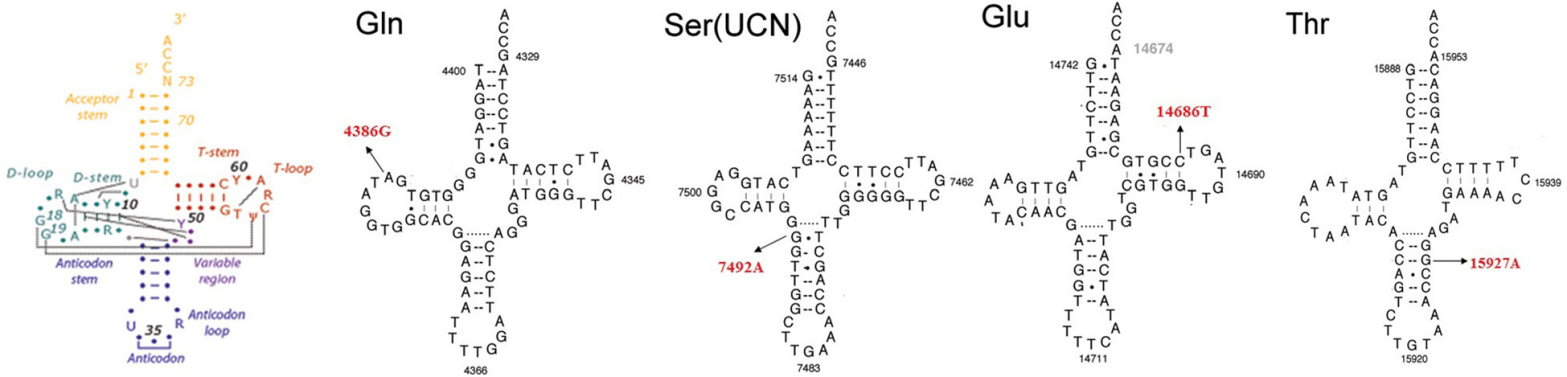
**Mitochondrial tRNA variants in subjects from nine Chinese Han pedigrees.** Cloverleaf structures of canonical tRNA and four mitochondrial tRNAs are shown. Arrows indicate the positions of the tRNA mutations.

To evaluate the effect of the variants on mitochondrial tRNA structure and function, we localized each variant to either the stem or loop of tRNA secondary structures. As shown in Figure 2, tRNA^Gln^ 4386 T > C was located in the loop, tRNA^Thr^ 15927G > A and tRNA^Ser(UCN)^ 7492 T > C were located in the anti-codon stem, and tRNA^Glu^ 14684G > A was located in the T-stem. In addition, a phylogenetic analysis was performed by comparing the human tRNA nucleotide variants with those in 16 other vertebrates. The CI was 31.2% in tRNA^Glu^ 14684G > A, 56.2% in tRNA^Thr^ 15927G > A, 62.5% in tRNA^Gln^ 4386 T > C and 81.2% in tRNA^Ser(UCN)^ 7492 T > C. tRNA^Glu^ 14684G > A was absent in healthy controls and in 2704 mtDNAs, tRNA^Ser(UCN)^ 7492 T > C was absent in the controls and was observed in <1% of 2704 mtDNAs, while tRNA^Thr^ 15927G > A and tRNA^Gln^ 4386 T > C were present in this control population and in >1% of 2704 mtDNAs. Based on these criteria, tRNA^Ser(UCN)^ 7492 T > C may have functional significance.

## Discussion

Maternal inheritance has several characteristics. Males and females inherit mitochondrial diseases equally, but always from their mother. A father cannot pass on mitochondrial disease to his children. In this study, we identified nine maternally inherited hypertension families from 108 hypertension individuals. Maternal influences on blood pressure can be explained by X-chromosomal inheritance, chromosomal imprinting [25], gestational mechanisms [26,27] and mitochondrial disorders [28]. For autosomal inheritance, morbidity in offspring of affected mothers should be equal to that in offspring of affected fathers. However, none of the offspring of affected fathers in these families had hypertension, so autosomal inheritance could be rejected. For X-linked inheritance, only females are affected, but all these families had both male and female patients, so X-linked inheritance could also be rejected. Gestational mechanisms could be excluded because the ratio of affected offspring from a hypertensive mother should be less than 50%; however, this ratio in all families was equal to or larger than 50%. Mitochondrial DNA abnormality is by far the most likely explanation for the etiology of hypertension in these pedigrees.

Essential HTN is a polygenic disease; however, efforts to identify genetic determinants of HTN have been directed primarily towards the nuclear genome, whereas the role of the mitochondrial genome remains relatively unexplored. Recently, we reported that mitochondrial DNA mutations contribute to HTN, including ND1 3308, tRNA^Met^ 4435, tRNA^Ile^ 4263, tRNA^Ile^ 4295 [11-15]. These studies focused on tRNA mutations. To identify further variants in the mitochondrial genome that contribute to HTN, we analyzed the entire mitochondrial genome (mtDNA) in hypertensive probands from families with typical maternally inherited hypertension. The entire human mitochondrial DNA sequence, of only 16 kb, has been mapped and encodes 13 proteins two rRNAs, and 22 tRNAs. In this study, we describe a total of 172 variants in the nine probands compared with the “Cambridge” reference sequence (CRS), including 77 variants in the D-loop, four in ND1, five in ND2, seven in COI, six in COII, two in ATPase8, 13 in ATPase6, three in COIII, four in ND3, one in ND4L, five in ND4, seven in ND5, four in ND6, and 13 in Cytb. In addition we found six variants in 12S rRNA, 11 in 16S rRNA and four in four tRNA genes (see Additional file 1 and Table 3). Of these 172 variants, 30 were not identified in the control subjects or in 2704 mtDNAs.

To investigate the contribution of these mutations to maternally inherited hypertension, we identified the 172 mutations in all maternally inherited members from the nine families. Remarkably, there were seven non-synonymous changes associated with amino acid substitutions, including four mutations in the ATP synthase F0 subunit 6, and three in cytochrome b. Among these mutations, ATPase_6_ 8794C > T is associated with exercise endurance/coronary atherosclerosis risk, [29,30] and Cytb 15662G > A is associated with complex mitochondriopathy [31]. The other five amino acid changes have not been previously reported. We also identified two mutations in 12S rRNA (1005 T > C and 1438A > G) and three in 16S rRNA (1736A > G, 1824 T > C and 2706A > G). tRNA^Ser(UCN)^ 7492C > T and tRNA^Thr^ 15927G > A presented in all maternal members of the probands. 12S rRNA 1005 T > C has been associated with deaf, [32] while 12S rRNA 1438A > G may be involved with schizophrenia, bipolar disorder, and major depressive disorder [33] The CI was 56.2% for tRNA^Thr^ 15927G > A and 81.2% for tRNA^Ser(UCN)^ 7492 T > C. tRNA^Ser(UCN)^ 7492 T > C was absent in the controls and present at <1% in 2704 mtDNAs, while tRNA^Thr^ 15927G > A was present in this control population and at >1% in 2704 mtDNAs. Based on these criteria, tRNA^Ser(UCN)^ 7492 T > C mutations may have functional significance.

## Conclusions

In this study, complete sequencing of the mitochondrial genome from nine maternally hypertensive probands allowed us to detect novel and previously unreported mtDNA variants. Several of the more common mutations have been previously reported to be associated with cardiovascular disease, such as 10398A > G, which is associated with end-stage renal disease in African Americans with HTN [34]. In summary, several mtDNA mutations may contribute to hypertension. In the future, additional mtDNA mutations may be discovered that could indicate candidate genes for hypertension. Thus, our findings provide new understanding of the pathophysiology of HTN and valuable information for the management and treatment of maternally inherited hypertension. For the members of these families with maternally inherited hypertension, we provided systematic follow-up and promoted secondary prevention, including risk factor control, use of medications, and self-management. Future research should further explore the emerging link between hypertension and mitochondrial dysfunction, and their cause/effect relationship.

## Additional file

Additional file 1:
**mtDNA variants in 9 Han Chinese probands with hypertension.**
