# Supplementary material for: Systematic analysis of the clinical and biochemical characteristics of maternally inherited hypertension in Chinese Han families associated with mitochondrial
Source: BMC Med Genomics. 2014 Dec 24;7:73. doi: 10.1186/s12920-014-0073-x (PMC4331388; doi:10.1186/s12920-014-0073-x)
Supplement: Additional file 1: — mtDNA variants in 9 Han Chinese probands with hypertension. [file 12920_2014_73_MOESM1_ESM.docx]

Supple 1 mtDNA variants in 9 Han Chinese probands with hypertension

| Gene | position | Nucleotide  change | Probands | Conservation  index (%)^a^ | WC  base-pairs^b^ | Conservation^c^ H/B/M/X | Amino acid change | Number of probands | See in 2704 mtDNAs^d^ | See in the controle |
| --- | --- | --- | --- | --- | --- | --- | --- | --- | --- | --- |
| D-loop | 73 | A to G | 5 |  |  |  |  |  | Yes | Yes |
|  | 146 | T to C | 1 |  |  |  |  |  | Yes | Yes |
|  | 150 | C to T | 1 |  |  |  |  |  | Yes | Yes |
|  | 198 | C to T | 1 |  |  |  |  |  | Yes | Yes |
|  | 199 | T to C | 1 |  |  |  |  |  | Yes | Yes |
|  | 200 | A to G | 1 |  |  |  |  |  | Yes | Yes |
|  | 215 | A to G | 1 |  |  |  |  |  | Yes | Yes |
|  | 235 | A to G | 1 |  |  |  |  |  | Yes | Yes |
|  | 239 | T to C | 1 |  |  |  |  |  | Yes | Yes |
|  | 263 | A to G | 6 |  |  |  |  |  | Yes | Yes |
|  | 276 | A to G | 1 |  |  |  |  |  | No | No |
|  | 280 | C to T | 1 |  |  |  |  |  | Yes | No |
|  | 310 | T to C | 3 |  |  |  |  |  | Yes | Yes |
|  | 317 | C to T | 1 |  |  |  |  |  | Yes | Yes |
|  | 318 | T to C | 1 |  |  |  |  |  | Yes | Yes |
|  | 320 | C to T | 1 |  |  |  |  |  | Yes | Yes |
|  | 326 | A to G | 1 |  |  |  |  |  | Yes | Yes |
|  | 343 | C to T | 1 |  |  |  |  |  | No | Yes |
|  | 376 | A to G | 1 |  |  |  |  |  | No | Yes |
|  | 382 | C to A | 1 |  |  |  |  |  | Yes | No |
|  | 385 | A to G | 1 |  |  |  |  |  | Yes | No |
|  | 390 | A to G | 1 |  |  |  |  |  | No | No |
|  | 400 | T to G | 1 |  |  |  |  |  | Yes | No |
|  | 404 | C to T | 1 |  |  |  |  |  | No | Yes |
|  | 408 | T to C | 1 |  |  |  |  |  | Yes | Yes |
|  | 419 | A to G | 1 |  |  |  |  |  | No | No |
|  | 420 | C to T | 1 |  |  |  |  |  | No | No |
|  | 426 | A to G | 1 |  |  |  |  |  | No | No |
|  | 432 | A to G | 1 |  |  |  |  |  | No | No |
|  | 441 | C to T | 1 |  |  |  |  |  | Yes | Yes |
|  | 446 | A to G | 1 |  |  |  |  |  | No | No |
|  | 470 | A to G | 1 |  |  |  |  |  | Yes | No |
|  | 473 | C to T | 2 |  |  |  |  |  | No | No |
|  | 476 | C to T | 2 |  |  |  |  |  | No | No |
|  | 481 | C to T | 2 |  |  |  |  |  | Yes | Yes |
|  | 483 | C to T | 1 |  |  |  |  |  | No | No |
|  | 486 | C to T | 1 |  |  |  |  |  | No | No |
|  | 489 | T to C | 4 |  |  |  |  |  | Yes | Yes |
|  | 491 | C to T | 1 |  |  |  |  |  | No | Yes |
|  | 496 | C to T | 1 |  |  |  |  |  | No | No |
|  | 497 | C to T | 1 |  |  |  |  |  | Yes | Yes |
|  | 505 | C to T | 1 |  |  |  |  |  | Yes | Yes |
|  | 506 | C to T | 2 |  |  |  |  |  | Yes | No |
|  | 527 | C to T | 1 |  |  |  |  |  | Yes | Yes |
|  | 530 | C to T | 1 |  |  |  |  |  | Yes | No |
|  | 549 | C to T | 1 |  |  |  |  |  | Yes | Yes |
|  | 554 | A to G | 1 |  |  |  |  |  | No | No |
|  | 558 | C to T | 1 |  |  |  |  |  | No | No |
|  | 565 | A to G | 1 |  |  |  |  |  | No | No |
|  | 567 | A to C | 2 |  |  |  |  |  | No | Yes |
|  | 574 | A to G | 1 |  |  |  |  |  | Yes | Yes |
| 12S rRNA | 709 | G to A | 1 | 64.3 | ↓G-C | G/A/A/— |  |  | Yes | Yes |
|  | 750 | A to G | 4 |  |  | A/A/G/- |  |  | Yes | Yes |
|  | 1005 | T to C | 1 |  |  | **T/T/T/T** |  |  | Yes | Yes |
|  | 1009 | C to T | 1 |  |  |  |  |  | Yes | No |
|  | 1438 | A to G | 8 | 100.0 | ↓A-U | A/A/A/G |  |  | Yes | Yes |
|  | 1598 | G to A | 1 |  |  | G/A/T/T |  |  | Yes | Yes |
| 16S rRNA | 1736 | A to G | 1 |  |  |  |  |  | Yes | Yes |
|  | 1824 | T to C | 1 |  |  |  |  |  | Yes | Yes |
|  | 2356 | A to G | 1 |  |  |  |  |  | Yes | Yes |
|  | 2448 | G to A | 1 |  |  |  |  |  | No | No |
|  | 2534 | G to A | 1 |  |  |  |  |  | No | No |
|  | 2673 | G to A | 1 |  |  |  |  |  | No | No |
|  | 2695 | G to A | 1 |  |  |  |  |  | No | No |
|  | 2706 | A to G | 9 |  |  | A/G/A/A |  |  | Yes | Yes |
|  | 2719 | G to A | 1 |  |  |  |  |  | No | No |
|  | 3010 | G to A | 1 |  |  | G/G/A/A |  |  | Yes | Yes |
|  | 3083 | T to C | 1 |  |  |  |  |  | Yes | Yes |
| ND1 | 3426 | A to G | 1 |  |  |  | syn |  | Yes | Yes |
|  | 3427 | G to A | 1 |  |  |  |  |  | No | Yes |
|  | 3970 | C to T | 1 |  |  |  | syn |  | Yes | Yes |
|  | 4140 | C to T | 1 |  |  |  | syn |  | Yes | Yes |
| tRNA^Gln^ | 4386 | T to C | 1 |  |  | T/T/A/C |  |  | Yes | Yes |
| ND2 | 4769 | A to G | 5 |  |  |  | syn |  | Yes | Yes |
|  | 4824 | A to G | 1 |  |  |  | Thr -> Ala |  | Yes | Yes |
|  | 4883 | C to T | 1 |  |  |  | syn |  | Yes | Yes |
|  | 5178 | C to A | 1 |  |  | L/T/T/T | Leu -> Met |  | Yes | Yes |
|  | 5277 | T to C | 1 |  |  |  |  |  | No | No |
| COI | 5969 | C to T | 1 |  |  |  |  |  | No | No |
|  | 6392 | T to C | 1 |  |  |  | syn |  | Yes | Yes |
|  | 6395 | C to T | 1 |  |  |  |  |  | No | No |
|  | 6531 | C to T | 1 |  |  |  | syn |  | Yes | No |
|  | 6689 | C to T | 1 |  |  |  | syn |  | Yes | No |
|  | 7028 | C to T | 7 |  |  |  | syn |  | Yes | Yes |
|  | 7250 | A to G | 1 |  |  |  | syn |  | Yes | No |
| tRNA ^Ser(UCN)^ | 7492 | C to T | 1 |  |  |  |  |  | Yes | No |
| COII | 7642 | G to A | 1 |  |  |  | syn |  | Yes | Yes |
|  | 7789 | G to A | 1 |  |  |  | syn |  | Yes | No |
|  | 7948 | C to T | 1 |  |  |  | syn |  | Yes | No |
|  | 8020 | G to A | 1 |  |  |  | syn |  | Yes | Yes |
|  | 8140 | C to T | 1 |  |  |  | syn |  | Yes | Yes |
|  | 8206 | G to A | 1 |  |  |  | syn |  | Yes | Yes |
| ATPase8 | 8413 | A to G | 2 |  |  |  | syn |  | Yes | Yes |
|  | 8414 | C to T | 1 |  |  |  | Leu -> Phe |  | Yes | Yes |
| ATPase6 | 8584 | G to A | 1 |  |  | A/V/V/I | Ala -> Thr |  | Yes | Yes |
|  | 8701 | A to G | 5 |  |  | T/S/L/Q | Thr -> Ala |  | Yes | Yes |
|  | 8791 | C to T | 1 |  |  |  |  |  | No | No |
|  | 8794 | C to T | 2 |  |  | H/H/H/Y | His -> Tyr |  | Yes | Yes |
|  | 8801 | T to C | 2 |  |  |  |  |  | No | Yes |
|  | 8803 | A to G | 1 |  |  |  | Thr -> Ala |  | Yes | Yes |
|  | 8805 | A to G | 2 |  |  |  | syn |  | Yes | Yes |
|  | 8808 | A to G | 1 |  |  |  |  |  | No | Yes |
|  | 8809 | A to G | 1 |  |  |  |  |  | No | Yes |
|  | 8810 | C to T | 1 |  |  |  |  |  | No | Yes |
|  | 8811 | C to T | 1 |  |  |  |  |  | No | No |
|  | 8860 | A to G | 3 |  |  | T/A/A/T | Thr -> Ala |  | Yes | Yes |
|  | 9099 | C to T | 1 |  |  |  | Ile -> Met |  | Yes | Yes |
| COIII | 9380 | G to A | 1 |  |  |  | syn |  | Yes | Yes |
|  | 9824 | T to C | 1 |  |  |  | syn |  | Yes | Yes |
|  | 9950 | T to C | 2 |  |  |  | syn |  | Yes | Yes |
| ND3 | 10172 | G to A | 1 |  |  |  | syn |  | Yes | No |
|  | 10181 | C to T | 1 |  |  |  | syn |  | Yes | No |
|  | 10398 | A to G | 6 |  |  | T/T/T/A | Thr -> Ala |  | Yes | Yes |
|  | 10400 | C to T | 4 |  |  |  | Thr -> Ala |  | Yes | Yes |
| ND4L | 10646 | G to A | 1 |  |  |  | syn |  | Yes | Yes |
| ND4 | 10873 | T to C | 5 |  |  |  | syn |  | Yes | Yes |
|  | 11440 | G to A | 1 |  |  |  | syn |  | Yes | Yes |
|  | 11665 | C to T | 1 |  |  |  | syn |  | Yes | No |
|  | 11719 | G to A | 6 |  |  |  | syn |  | Yes | Yes |
|  | 11969 | G to A | 1 |  |  | A/A/G/A | Ala -> Thr |  | Yes | Yes |
| ND5 | 12361 | A to G | 1 |  |  | T/L/L/L | Thr -> Ala |  | Yes | Yes |
|  | 12549 | C to T | 1 |  |  |  | syn |  | Yes | Yes |
|  | 12705 | C to T | 4 |  |  |  | syn |  | Yes | Yes |
|  | 13135 | G to A | 1 |  |  | A/T/T/M | Ala -> Thr |  | Yes | Yes |
|  | 13152 | A to G | 1 |  |  |  | syn |  | Yes | Yes |
|  | 13821 | C to T | 1 |  |  |  |  |  | No | No |
|  | 14122 | A to G | 1 |  |  |  |  |  | No | No |
| ND6 | 14318 | T to C | 1 |  |  | N/N/D/S | Asn -> Ser |  | Yes | Yes |
|  | 14337 | C to T | 1 |  |  |  | Val -> Met |  | Yes | No |
|  | 14502 | T to C | 1 |  |  | I/I/I/I | Ile -> Val |  | Yes | Yes |
|  | 14668 | C to T | 1 |  |  |  | syn |  | Yes | Yes |
| tRNA ^Glu^ | 14686 | G to A | 1 |  |  |  |  |  | No | No |
| Cytb | 15040 | C to T | 1 |  |  |  | syn |  | Yes | Yes |
|  | 15043 | G to A | 1 |  |  |  | syn |  | Yes | Yes |
|  | 15071 | T to C | 1 |  |  | Y/F/F/Y | Tyr -> His |  | Yes | Yes |
|  | 15218 | A to G | 1 |  |  | T/T/T/N | Thr -> Ala |  | Yes | Yes |
|  | 15223 | C to T | 1 |  |  |  | syn |  | Yes | No |
|  | 15301 | G to A | 1 |  |  |  | syn |  | Yes | Yes |
|  | 15326 | A to G | 4 |  |  | T/M/I/I | Thr -> Al |  | Yes | Yes |
|  | 15379 | C to T | 1 |  |  |  | syn |  | Yes | No |
|  | 15427 | A to G | 1 |  |  |  | syn |  | Yes | No |
|  | 15508 | C to T | 1 |  |  |  | syn |  | Yes | Yes |
|  | 15662 | A to G | 1 |  |  | I/L/F/L | Ile -> Val |  | Yes | Yes |
|  | 15850 | T to C | 1 |  |  |  | syn |  | Yes | Yes |
|  | 15851 | A to G | 1 |  |  | I/A/S/M | Ile -> Val |  | Yes | Yes |
| tRNA ^Thr^ | 15927 | G to A | 1 |  |  | G/G/G/G |  |  | Yes | Yes |
| D-loop | 16025 | T to C | 1 |  |  |  |  |  | No | Yes |
|  | 16093 | T to C | 2 |  |  |  |  |  | Yes | Yes |
|  | 16100 | A to G | 1 |  |  |  |  |  | No | No |
|  | 16102 | T to C | 1 |  |  |  |  |  | Yes | No |
|  | 16111 | C to T | 1 |  |  |  |  |  | Yes | Yes |
|  | 16129 | G to A | 2 |  |  |  |  |  | Yes | Yes |
|  | 16140 | T to C | 1 |  |  |  |  |  | Yes | Yes |
|  | 16166 | A to G | 2 |  |  |  |  |  | Yes | Yes |
|  | 16172 | T to C | 1 |  |  |  |  |  | Yes | Yes |
|  | 16182 | A to G | 1 |  |  |  |  |  | Yes | Yes |
|  | 16183 | A to G | 2 |  |  |  |  |  | Yes | Yes |
|  | 16189 | T to C | 2 |  |  |  |  |  | Yes | Yes |
|  | 16194 | A to G | 1 |  |  |  |  |  | Yes | Yes |
|  | 16202 | A to G | 1 |  |  |  |  |  | No | No |
|  | 16223 | C to T | 3 |  |  |  |  |  | Yes | Yes |
|  | 16244 | G to A | 1 |  |  |  |  |  | Yes | Yes |
|  | 16278 | C to T | 1 |  |  |  |  |  | Yes | Yes |
|  | 16288 | T to C | 1 |  |  |  |  |  | Yes | Yes |
|  | 16295 | C to T | 1 |  |  |  |  |  | Yes | Yes |
|  | 16298 | T to C | 1 |  |  |  |  |  | Yes | Yes |
|  | 16311 | T to C | 2 |  |  |  |  |  | Yes | Yes |
|  | 16357 | T to C | 1 |  |  |  |  |  | Yes | Yes |
|  | 16362 | T to C | 1 |  |  |  |  |  | Yes | Yes |
|  | 16482 | A to G | 1 |  |  |  |  |  | Yes | Yes |
|  | 16497 | A to G | 1 |  |  |  |  |  | Yes | Yes |
|  | 16519 | T to C | 3 |  |  |  |  |  | Yes | Yes |

^a^ The conservation index (CI) was then defined as the percentage of the human nucleotide variants with other 16 vertebrates that have the wild-type nucleotide at that position.

^b^ Classic Watson–Crick (WC) base-pair: created (↑) or abolished (↓).

^c^ Conservation of amino acid for polypeptides or nucleotide for rRNAs, in human (H), mouse (M), bovine (B), and Xenopus laevis (X).

^d^ See http://www.genpat.uu.se/mtDB

|  |  |  |  |
| --- | --- | --- | --- |
|  |  |  |  |
|  |  |  |  |
|  |  |  |  |
|  |  |  |  |
|  |  |  |  |
|  |  |  |  |
|  |  |  |  |
|  |  |  |  |
|  |  |  |  |
